# Klippel-Feil syndrome at neonatal age presentation: rare case report

**DOI:** 10.1097/MS9.0000000000003574

**Published:** 2025-07-16

**Authors:** Hansa Haftu Lemma, Hailemariam Gebrearegay, Hiwet Aregawi Lemma

**Affiliations:** a Child Health Department, College of Health Science, Mekelle University, Tigray, Ethiopia; b Internal Medicine Department, College of Medicine and Health Science, Adigrat University, Tigray, Ethiopia

**Keywords:** case report, Klippel-Feil syndrome, neonates, Tigray

## Abstract

**Background:**

Klippel-Feil syndrome (KFS) is a rare, heterogeneous, congenital disorder of the cervical spine due to failure of segmentation during early gestation. We will present a neonate with KFS who had a classic triad.

**Case presentation:**

A 5-month-old female infant, now in regular orthopedic and pediatric follow-up, was admitted to the neonatal intensive care unit (NICU) immediately after delivery (at the age of 1 hour) with fast breathing and low oxygen saturation. She had a short neck, low hairline, and severely limited neck mobility. A neck X-ray revealed the cervical spine as a single, small bone, and a chest X-ray also showed a left lung field opacity and multiple infiltrations. Abdominal U/S showed left-sided hydronephrosis, and echocardiography revealed the presence of a patent ductus arteriosus (PDA) and an atrial septal defect (ASD). She was treated for neonatal sepsis with antibiotics, oxygen, nasogastric feeding, and showed significant improvement in his distress. After Orthopedics was consulted, she was discharged with regular follow-up appointments to both pediatric (cardiac and renal) and orthopedic for further care.

**Clinical discussion:**

Different literature mentions various causes of short neck in neonates. KFS is rare and the diagnosis of KFS requires a holistic approach and a high index of suspicion to exclude other causes of a short neck, especially in the absence of a genetic test.

**Conclusion:**

Even though a genetic test is mandatory to confirm the diagnosis and the subtypes, in limited settings like our case, clinical and radiological evidence may help to reach a diagnosis.

## Introduction

Klippel-Feil syndrome (KFS) is a heterogeneous congenital cervical spine disorder characterized by the fusion of two or more cervical vertebrae, resulting from a failure of segmentation along the embryo’s developing axis during critical fetal development^[^1,2^]^. KFS occurs in approximately 1 in 40 000 to 42 000 live births, with a higher prevalence in females (60%). Although the age at presentation can vary depending on the extent of the fusion, it may be diagnosed in utero or immediately after delivery^[^2^]^. Patients may exhibit the classic triad of manifestations—short neck, low hairline, and limited neck movement—though only about 50% present with all three typical features. Additionally, patients may have associated anomalies affecting other organ systems, including the renal, cardiac, and nervous systems^[^2^]^.

**HIGHLIGHTS**

- Klippel-Feil syndrome (KFS) is a rare case, one in 42 000.
- KFS diagnosed at neonatal age.
- KFS was diagnosed clinically with the classic triads (short neck, low hairline, and limited neck movement).

There are different types of KFS based on the mode of inheritance and the specific region of vertebral synostosis, which may correlate with the severity of the condition^[^3^]^. According to the classification, the three types of KFS are: Type 1, Type 2, and Type 3^[^4^]^. The age at diagnosis of KFS can vary, with cases identified in utero^[^5^]^, during childhood^[^1,6^]^, or in adulthood. These variations may be related to the specific type of KFS and any associated anomalies.

Our primary objective is to report a rare case of this musculoskeletal disorder in a neonate diagnosed with KFS, who presents with the classic clinical triad, significant radiologic findings, and associated anomalies.

## Case report

RB is a 5-month-old female infant currently on regular follow-up at orthopedic and high-risk pediatric clinics (cardiac and renal), following an admission to the NICU at 1 hour of age. She presented with fast breathing, grunting, and low oxygen saturation. The family also reported a short neck, low hairline, and difficulty moving her neck. She was born to a 33-year-old para-three mother via cesarean section, indicated by a prior uterine scar and premature rupture of membranes. There was no history of consanguinity or similar illness in the family. No limb or lip discoloration was observed.

**Figure 1.**
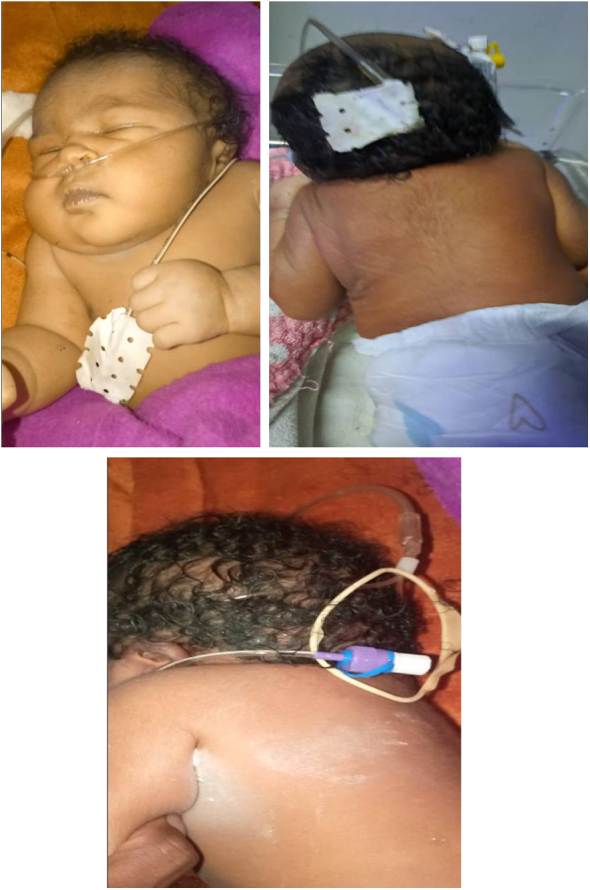
Physical findings (invisible neck, low hairline) in different views of the patient with Klippel-Feil syndrome.

**Physical Examination**: Pulse rate (PR): 140 bpm, respiratory rate (RR): 67 breaths/min, temperature (T): 36.7°C, weight: 3.4 kg, head circumference (HC): 38 cm, length: 49 cm, SpO_2_: 85% on room air (98% with supplemental oxygen). She had an extremely short, almost invisible neck, a low posterior hairline, and severely restricted neck movement (Fig. 1). Signs of respiratory distress were evident, including chest indrawing, intercostal retractions, nasal flaring, and decreased air entry in the posterior left chest. A holosystolic murmur was heard in the left upper chest without other added sounds. Investigations: CBC: Leukocytosis with a left shift, Echocardiography: Patent ductus arteriosus (PDA) and atrial septal defect (ASD), Neck X-ray: Cervical vertebrae appeared as a single small bone, indicating complete cervical fusion

**Figure 2.**
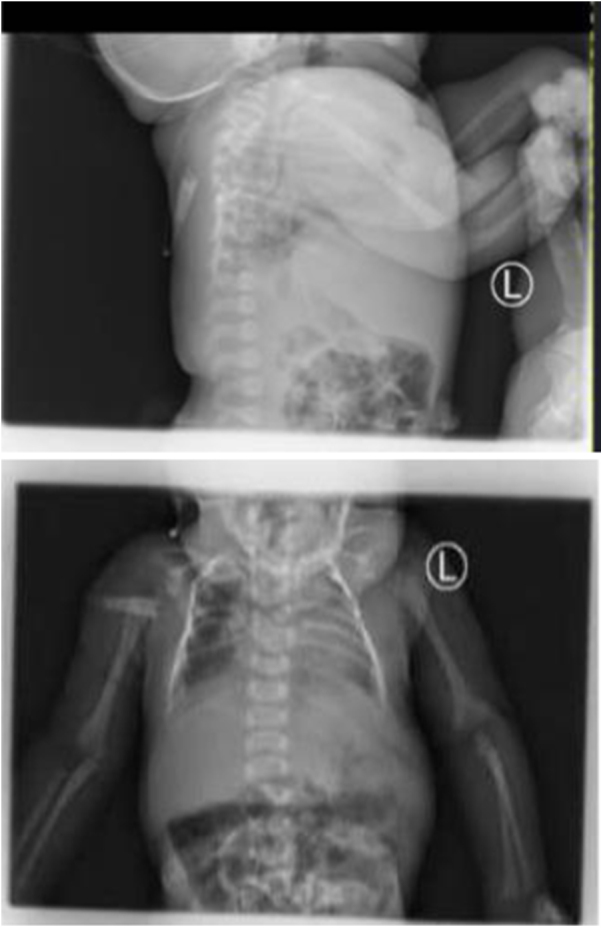
Chest and neck X-ray of the patient with Klipple-Feil syndrome showed complete cervical bone fusion, and the upper thoracic vertebra is also fused.

Chest X-ray (PA and lateral): Opacity and diffuse infiltration, mainly in the left lung field and upper thoracic fusion (Fig. 2), Neck ultrasound: No neck space available for the probe to assess cervical vertebrae, Abdominal ultrasound: Left-sided hydronephrosis, other tests (blood culture, cranial ultrasound): Normal. She was diagnosed with neonatal sepsis and Klippel-Feil syndrome (KFS). Due to the unavailability of genetic testing, the diagnosis was made clinically based on the presence of the classic clinical triad and corroborating radiologic findings, along with associated anomalies. Management: She was treated with intravenous antibiotics—ampicillin (100 mg/kg, twice daily) and gentamicin (5 mg/kg/day) for 10 days—along with oxygen therapy and nasogastric feeding. The orthopedic team was consulted and recommended regular follow-up. After a 20-day NICU stay, the infant improved and was discharged with scheduled follow-up appointments in pediatric (renal and cardiac) and orthopedic clinics.

## Discussion

Klippel-Feil syndrome (KFS) was first described in 1912 by Klippel and Feil. It is a rare, heterogeneous skeletal disorder primarily involving fusion of the cervical vertebrae, often accompanied by anomalies in other organ systems. These associated anomalies can include spinal abnormalities such as scoliosis, facial dysmorphisms, and involvement of visceral organs, particularly the kidneys and heart. Although it is uncommon for patients to exhibit the full spectrum of findings, the classic triad of KFS includes a short neck, low hairline, and limited neck movement^[^1,3^]^. In our patient, all components of the classic triad were observed, along with radiological evidence of cervical fusion and associated renal and cardiac anomalies. While she has not yet developed complications involving other systems, close monitoring and multidisciplinary evaluation are essential. The exact etiology of KFS remains unclear; however, both genetic and environmental factors are believed to contribute to its development^[^5^]^.

Other differential diagnoses for a short neck in neonates include Noonan syndrome, congenital muscular torticollis (CMT), and skeletal dysplasia. Noonan syndrome may present with a short neck, low hairline, and congenital heart defects—features also seen in our patient. However, our patient lacks the characteristic craniofacial abnormalities, bleeding tendencies, and chest deformities typically associated with Noonan syndrome^[^7^]^. Furthermore, radiologic evidence of cervical fusion supports the diagnosis of KFS over Noonan syndrome. Another potential differential diagnosis is bilateral congenital muscular torticollis (CMT), which can present with a short, tight neck, lower hairline, and limited neck movement. However, CMT is usually unilateral and associated with facial asymmetry. Additionally, CMT does not typically involve organ anomalies or cervical vertebral fusion. In our patient, the presence of a truly short and nearly invisible neck—not merely a tight one—along with associated cardiac and renal anomalies and radiographic confirmation of cervical vertebral fusion, strongly supports the diagnosis of KFS^[^8^]^.

Most cases of KFS are sporadic, though familial inheritance patterns—including autosomal dominant, autosomal recessive, and X-linked—have been reported in those with a positive family history, depending on the subtype^[^5,6^]^. Radiographic evaluation of the cervical spine in infants and young children can be challenging due to immature skeletal structures^[^9^]^. Similar to previously reported cases and case series, our patient exhibited the classic clinical triad—short neck, low hairline, and severely restricted neck movement—as well as cardiac (ASD and PDA) and renal (left-sided hydronephrosis) anomalies. She had no neurological deficits or limb deformities, and there was no family history of similar illness or parental consanguinity.

The timing of diagnosis depends on the type and extent of vertebral fusion. KFS is classified into three types^[^10^]^: **Type I:** Extensive fusion of cervical vertebrae, **Type II**: Fusion of one or two cervical vertebrae in a non-contiguous pattern, and **Type III**: Cervical fusion associated with fusion of thoracic or lumbar vertebrae. Based on imaging findings, our patient likely falls into **Type III**, given the complete cervical fusion and involvement of the upper thoracic vertebrae. Patients with significant cervical fusion are more likely to present early, either in utero or during the neonatal period. In contrast, milder cases may remain undiagnosed until adolescence or adulthood. A higher incidence has been reported in females, regardless of the age at diagnosis^[^1,5^]^. In our case, the patient is female and was diagnosed during the neonatal period, which supports the likelihood of a more severe form.

Despite her admission for neonatal sepsis, she had no neurological deficits; she was active, with normal movement in all extremities, and no pain. She was assessed by both a pediatric neurologist and an orthopedic specialist. As she remained neurologically asymptomatic, she was discharged with a plan for conservative management and regular follow-up in cardiac, renal, and orthopedic clinics to monitor for potential complications and consider surgical intervention if needed.

## Conclusion

Despite its rare occurrence, KFS may present in the classic triads in around 50%, and patients should be evaluated for other associated anomalies for better early detection and management.

### Strengths and limitations

The strength of this case report lies in the timely diagnosis of KFS during the neonatal age. Such cases are rare and often missed. The progressive follow-up is important for regular monitoring of any complications that may develop related to the spinal cord and other neurologic dysfunctions. The main limitation is the inability to confirm the diagnosis with a genetic test due to its unavailability, and the test will be done at the time of its availability.

## Data Availability

The datasets are available from the corresponding author upon reasonable request.

## Research registration unique identifying number (UIN)

The research is registered with a unique identifying number of researchregistry11141 (https://www.researchregistry.com/browse-theregistry#home/).
